# RNA Secondary Structure-Based Design of Antisense Peptide Nucleic Acids for Modulating Disease-Associated Aberrant Tau Pre-mRNA Alternative Splicing

**DOI:** 10.3390/molecules24163020

**Published:** 2019-08-20

**Authors:** Alan Ann Lerk Ong, Jiazi Tan, Malini Bhadra, Clément Dezanet, Kiran M. Patil, Mei Sian Chong, Ryszard Kierzek, Jean-Luc Decout, Xavier Roca, Gang Chen

**Affiliations:** 1 NTU Institute for Health Technologies (HeathTech NTU), Interdisciplinary Graduate School, Nanyang Technological University, 50 Nanyang Drive, Singapore 637553, Singapore; 2 Division of Chemistry and Biological Chemistry, School of Physical and Mathematical Sciences, Nanyang Technological University, 21 Nanyang Link, Singapore 637371, Singapore; 3 School of Biological Sciences, Nanyang Technological University, Singapore 637551, Singapore; 4 University Grenoble Alpes/CNRS, Département de Pharmacochimie Moléculaire, ICMG FR 2607, UMR 5063, 470 Rue de la Chimie, F-38041 Grenoble, France; 5 Geriatric Education & Research Institute, 2 Yishun Central 2, Singapore 768024, Singapore; 6 Institute of Bioorganic Chemistry, Polish Academy of Sciences, Noskowskiego 12/14, 61-704 Poznan, Poland

**Keywords:** RNA structure, strand invasion, antisense, PNA, exon skipping, exon inclusion

## Abstract

Alternative splicing of tau pre-mRNA is regulated by a 5′ splice site (5′ss) hairpin present at the exon 10–intron 10 junction. Single mutations within the hairpin sequence alter hairpin structural stability and/or the binding of splicing factors, resulting in disease-causing aberrant splicing of exon 10. The hairpin structure contains about seven stably formed base pairs and thus may be suitable for targeting through antisense strands. Here, we used antisense peptide nucleic acids (asPNAs) to probe and target the tau pre-mRNA exon 10 5′ss hairpin structure through strand invasion. We characterized by electrophoretic mobility shift assay the binding of the designed asPNAs to model tau splice site hairpins. The relatively short (10–15 mer) asPNAs showed nanomolar binding to wild-type hairpins as well as a disease-causing mutant hairpin C+19G, albeit with reduced binding strength. Thus, the structural stabilizing effect of C+19G mutation could be revealed by asPNA binding. In addition, our cell culture minigene splicing assay data revealed that application of an asPNA targeting the 3′ arm of the hairpin resulted in an increased exon 10 inclusion level for the disease-associated mutant C+19G, probably by exposing the 5′ss as well as inhibiting the binding of protein factors to the intronic spicing silencer. On the contrary, the application of asPNAs targeting the 5′ arm of the hairpin caused an increased exon 10 exclusion for a disease-associated mutant C+14U, mainly by blocking the 5′ss. PNAs could enter cells through conjugation with amino sugar neamine or by cotransfection with minigene plasmids using a commercially available transfection reagent.

## 1. Introduction

Tauopathies are a class of neurodegenerative disorders characterized by the formation of neurofibrillary tangles and paired helical filaments composed of microtubule-associated protein tau (MAPT) [1,2,3,4,5]. Tauopathies include Pick′s disease, Alzheimer′s disease, as well as frontotemporal dementia and parkinsonism linked to chromosome 17 (FTDP-17) [3]. FTDP-17 is an autosomal dominant neurodegenerative disorder that includes behavioral and personality changes, cognitive impairment, and motor symptoms [6]. FTDP-17 is caused by mutations in the *MAPT* gene, which encodes the tau protein [7,8,9]. Tau proteins are predominantly expressed in neurons and are involved in microtubule assembly, morphogenesis, neuron cytoskeletal maintenance, and axonal transport [10,11].

**Figure 1:**
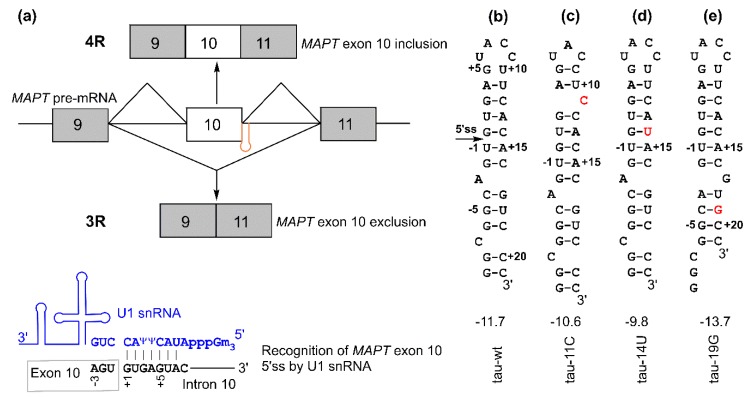
Microtubule-associated protein tau (*MAPT*) exon 10 5′ splice site recognition: (**a**) (top) schematic of *MAPT* pre-mRNA containing exon 9 (gray box), exon 10 (white box), and exon 11 (gray box). The regulatory hairpin (shown in orange) is located at the junction of exon 10 and intron 10, and 4R and 3R isoforms are generated based on the inclusion or exclusion (skipping) of *MAPT* exon 10. (bottom) Schematic of the recognition of the 5′ splice site by U1 small nuclear RNA (snRNA) (shown in blue). (**b**–**e**) Secondary structures for tau pre-mRNA exon 10 splice site hairpins. The values shown below the structures are folding free energies (in kcal/mol, at 1 M NaCl, pH 7.0) predicted by RNAstructure program [29,30]. The disease-causing mutations are shown in red. The +11C and +19G mutations result in structural rearrangement in the top and bottom stems, respectively [29]. The 5′ splice site located at the 5′ arm of the hairpin is indicated with an arrow in panel (b). The 5′ and 3′ arms of the hairpin may contain the exonic splicing enhancer elements and intronic splicing silencer/modulator sequences, respectively, as potential binding sites of *trans*-acting protein factors [31,32,33,34].

The *MAPT* gene contains 16 exons, with exons 2, 3, and 10 alternatively spliced to generate six tau isoforms. Alternative splicing of exon 10 gives rise to tau isoforms with four microtubule-binding repeat domains (4Rs) upon exon 10 inclusion or three repeats (3Rs) upon exon skipping (exclusion) (Figure 1a). The ratio of 4R/3R isoforms is maintained at close to 1:1 [7,12,13,14]. Either 3R or 4R isoforms or both can be present in tau protein filaments [4,5,7,15,16,17].

An RNA hairpin structure at the tau pre-mRNA exon 10 5′ splice site (5′ss, located at the 3′ end of exon 10 and the 5′ end of intron 10, Figure 1) may regulate the alternative splicing of exon 10 and thus the ratio of 4R/3R isoforms [18,19,20,21,22,23,24]. The formation of this hairpin masks the 5′ss, thus inhibiting its recognition by U1 small nuclear ribonucleoprotein (U1 snRNP, Figure 1a), which is a key initial step in pre-mRNA splicing [25,26,27]. Point mutations in the hairpin region affect its stability by introducing mismatched base pairs or by structural rearrangement within the hairpin [15,18,19,20,21,22,23,28,29].

**Figure 2:**
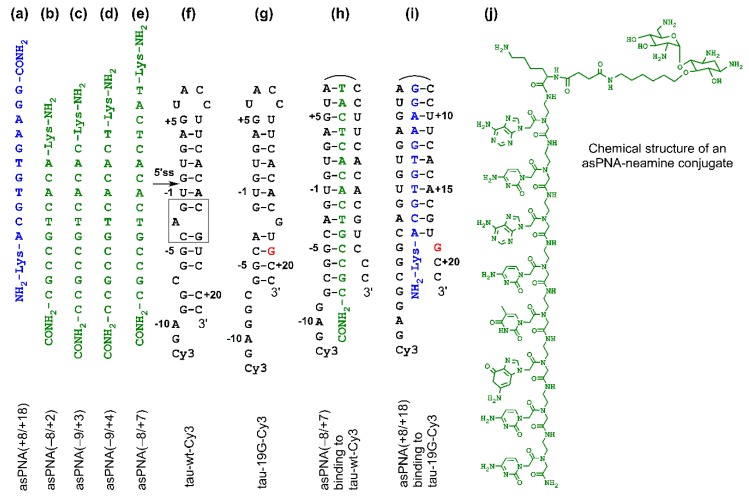
Sequences and structures of PNAs and RNAs. (**a**–**e**) PNAs studied in this paper. (**f**,**g**) Cy3-labeled tau pre-mRNA wild-type and mutant +19G RNA hairpin constructs used for nondenaturing PAGE assay. The gray box represents the A bulge structure, resulting in the formation of top and bottom stems. (**h**) A complex formed between antisense PNA asPNA(−8/+7) and hairpin tau-wt-Cy3. (**i**) A complex formed between asPNA(+8/+18) and hairpin tau-19G-Cy3. (**j**) Chemical structure of a PNA–neamine conjugate. The PNA is an 8-mer and is shown for illustration purposes. The Lys residue (attached with neamine) has an L configuration.

The hairpin has a single A bulge, which results in the formation of a top stem and a bottom stem above and below the A bulge, respectively (Figure 1b and Figure 2f). Mutations in the relatively more stable top stem often destabilize it by introducing a mismatched base pair or by local structural rearrangement (Figure 1c,d) and tend to increase the 4R/3R ratio [15,29]. On the other hand, a single C-to-G mutation in the relatively less stable bottom stem at the 19th nucleotide downstream of the 5′ss (C+19G or +19G) (Figure 1e) causes a significant decrease in the 4R/3R ratio [15,29,33]. The +19G mutation alters the structure of the bottom stem, resulting in the formation of a new bottom stem with enhanced stability (Figure 1e and Figure 2g) [29]. Abnormal 4R/3R ratios caused by these mutations lead to the pathogenesis of FTDP-17. Thus, this particular RNA hairpin becomes an important target in the development of therapies for FTDP-17. As exemplified by the recent US Food and Drug Administration (FDA)-approved drug nusinersen (Spinraza) for spinal muscular atrophy [35], splice-switching antisense oligonucleotides (SSOs) are promising therapeutic agents for neurodegenerative and other diseases [36,37]. SSOs have been utilized for targeting the tau pre-mRNA hairpin region for the regulation of the 4R/3R ratio in vitro and in vivo [16,38,39,40,41,42].

Peptide nucleic acid (PNA) was first introduced by Nielsen and his coworkers in 1991 [43]. Unlike natural nucleic acids (DNA and RNA), a canonical PNA contains a neutral *N*-(2-aminoethyl)-glycine (AEG) backbone, a methylene carbonyl linker, and nucleobases (Figure 2) [44]. The neutral PNA backbone results in no electrostatic repulsion upon hybridization with negatively charged RNA and DNA strands [45,46]. Thus, compared to Watson–Crick duplexes containing DNA and RNA strands, PNA–DNA or PNA–RNA Watson–Crick duplexes show enhanced stabilities [47]. Strong hybridization of PNA to the complementary RNA/DNA may allow the strand invasion of preformed duplex structures of RNA and DNA [48]. In addition, compared to unmodified RNA and DNA, PNA has several advantages in that it is resistant against nucleases and proteases and is immunologically inert [49,50,51,52]. PNAs have been utilized for replication inhibition, genome editing, transcription arrest, splicing correction, translation arrest, and noncoding RNA function regulation [44,53,54,55,56,57,58,59,60,61,62,63,64,65].

The tau pre-mRNA exon 10 splice site (Figure 1) has a relatively short stem interrupted by an A bulge and other non-Watson–Crick structures, which may allow for invasion by PNAs. Here, we characterized the binding of a series of antisense PNAs (asPNAs) to tau pre-mRNA exon 10 5′ss hairpin structures through strand invasion. In addition, we carried out a cell culture minigene splicing assay for asPNAs conjugated with neamine or cotransfected with minigene plasmids.

## 2. Results and Discussion

### 2.1. asPNAs Can Invade Tau Pre-mRNA Hairpin

We made asPNAs that are complementary to the 5′ arm or 3′ arm of the tau pre-mRNA exon 10 5′ss hairpin (Figure 2a–e). The formation of a stable PNA–RNA duplex targeting the 3′ arm of the hairpin was expected to expose the 5′ss and increase the exon 10 inclusion level (Figure 2i). On the contrary, formation of a PNA–RNA duplex targeting the 5′ arm of the hairpin was expected to block the 5′ss and increase exon 10 skipping (exclusion) (Figure 2h).

**Figure 3:**
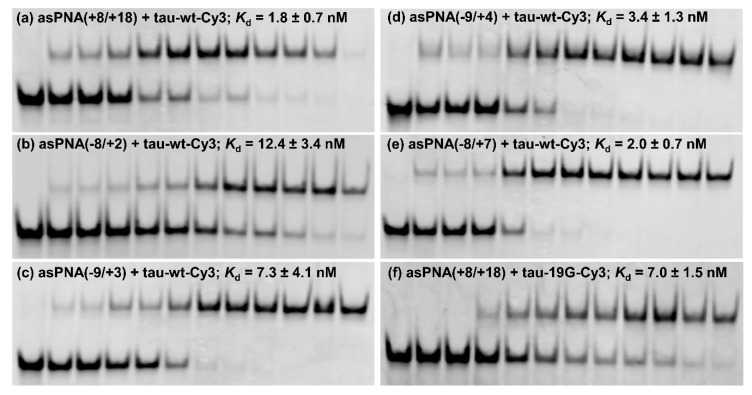
Nondenaturing PAGE study of various asPNAs binding to tau-wt-Cy3 and tau-19G-Cy3 (see Figure 2f,g). The gels contained a running buffer of 1× TBE, pH 8.3, and were run for 5 h at 250 V. The incubation buffer was 200 mM NaCl, 0.5 mM EDTA, and 20 mM HEPES at pH 7.5. RNA hairpins were loaded at 5 nM in 20 µL. The PNA concentrations in the lanes from left to right were 0, 0.5, 1, 2, 5, 10, 15, 20, 30, 50, 100, and 200 nM, respectively. (**a**,**f**) asPNAs binding to the 3′ arm of the splice site hairpins. Compared to tau-WT-Cy3, tau-19G-Cy3 showed weakened binding to asPNA(+8/+18), even though the C+19G mutation was adjacent to, but not within, the recognition site of the asPNA (Figure 2), indicating that the single C+19G mutation stabilized the splice site hairpin. (**b**–**e**) asPNAs binding to the 5′ arm of the splice site hairpin.

We made an 11-mer PNA (asPNA(+8/+18), NH_2_-Lys-ACGTGTGAAGG-CONH_2_, Figure 2a), which was complementary to the 3′ arm of the RNA hairpin. Our nondenaturing PAGE data revealed that asPNA(+8/+18) bound tightly to the Cy3-labeled wild-type tau pre-mRNA hairpin (tau-wt-Cy3, *K*_d_ = 1.8 ± 0.7 nM) in a near physiological buffer (200 mM NaCl, pH 7.5) (Figure 3a, Supplementary Figure S2). Remarkably, asPNA(+8/+18) showed a weakened binding to the hairpin with a +19G mutation (tau-19G-Cy3, *K*_d_ = 7.0 ± 1.5 nM) (Figure 3f, Supplementary Figure S2), even though the mutation was adjacent to, but not within, the recognition site of the asPNA. A C+19G mutation has been shown to cause RNA secondary structural rearrangement, resulting in the stabilization of the splice site hairpin (Figure 1e and Figure 2f) [29]. In addition, asPNA(+8/+18) may have invaded two and one base pairs below the A bulge and G bulge in the wild-type and +19G mutant, respectively (Figure 2). Clearly, a +19G mutation causes the formation of a stabilized stem below the G bulge, which in turn results in the stabilization of the top stem above the G bulge and reduces the invasion by asPNA(+8/+18). Thus, the strand invasion of RNA structures by asPNA may be used to reveal the structural stability changes of target RNA hairpins upon subtle single mutations.

We next made a 15-mer PNA (asPNA(−8/+7), NH_2_-Lys-TACTCACACTGCCGC-CONH_2_, Figure 2e), which is complementary to the 5′ arm of the RNA hairpin. Our nondenaturing PAGE data revealed that asPNA(−8/+7) showed strong binding to hairpin tau-wt-Cy3 (*K*_d_ = 1.8 ± 0.7 nM, 200 mM NaCl, pH 7.5) (Figure 3e, Supplementary Figure S2). Shortening the asPNA length resulted in the weakening of the binding (asPNA(−9/+4), 13-mer, *K*_d_ = 3.4 ± 1.3 nM; asPNA(−9/+3), 12-mer, *K*_d_ = 7.3 ± 4.1 nM; and asPNA(−8/+2), 10-mer, *K*_d_ = 12.4 ± 3.4 nM) (Figure 2b–d and Figure 3b–d, Supplementary Figure S2). The top stem of the hairpin is relatively stable, as revealed by our previous bulk thermal melting and single-molecule mechanical unfolding studies [29]. Consistently, lengthening the asPNAs to invade the top stem above the A bulge (e.g., asPNA(−8/+2) versus asPNA(−8/+7)) resulted in a relatively moderate enhancement in binding. The relatively narrow range of *K*_d_ values may be consistent with the fact that asPNAs bind to the tau pre-mRNA hairpin through the disruption (invasion) of preformed RNA structures. For example, upon the binding of asPNA(−8/+2), a hairpin structure involving the RNA residues from +3 to +12 (see Figure 2f) may still form, with the remaining RNA stem coaxially stacked on the PNA–RNA duplex. However, upon the binding of asPNA(−8/+7), the tau pre-mRNA hairpin is completely disrupted (Figure 2h). Thus, depending on the target RNA structure and final asPNA-bound complex structure, lengthening an RNA structure-disrupting asPNA may result in a small net enhancement in binding free energy. Further experiments are required to understand the binding properties for asPNAs targeting structured RNAs.

PNAs are able to invade certain DNA duplexes [66,67,68,69]. We tested the binding of asPNA(−8/+7) to the model tau wild-type DNA duplex (tau-wt-DNA), which encodes the splice site hairpin of tau pre-mRNA (Supplementary Figure S3a–c). Our nondenaturing PAGE data revealed that asPNA(−8/+7) showed no binding to the fully complementary tau model DNA duplex encoding the tau pre-mRNA hairpin sequence (Supplementary Figure S3d), probably because the targeted region is relatively G-C pair rich and is in the middle of a duplex [67,69]. Thus, the A bulge structure and other non-Watson–Crick structures destabilize the tau pre-mRNA hairpin and facilitate the invasion of asPNAs and other antisense strands [16,42].

### 2.2. asPNAs Can Alter Tau Minigene Pre-mRNA Splicing in Cell Cultures

We tested the cell culture activities of the asPNAs in modulating the tau pre-mRNA minigene splicing for the +19G and +14U mutants, which exhibited overly enhanced exclusion and inclusion of exon 10, respectively. It has been previously reported that PNAs may be delivered into cells by incorporating PNAs into liposome structures [70]. In our study, HEK293T cells were cotransfected with the minigenes and asPNAs using the commercially available X-tremeGENE 9 DNA Transfection Reagent (a nonliposomal multicomponent reagent, method A).

**Figure 4:**
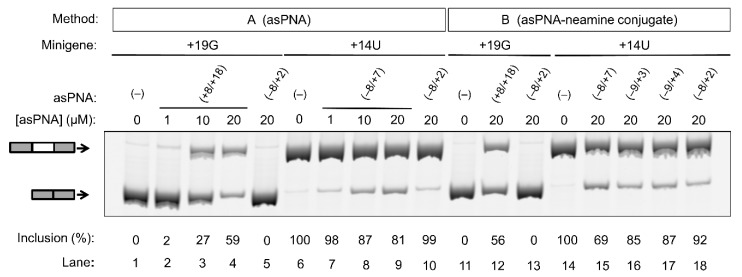
Effect of asPNAs on tau pre-mRNA exon 10 splicing. Representative RT-PCR data of the cell culture splicing assays are shown. The levels of exon 10 inclusion were derived from three experimental replicates (samples from independent minigene transfections, with the standard deviations <10%). In method A, the PNAs were mixed with the minigene transfection mixture and incubated for 20 min prior to transfection. In method B, the PNA–neamine conjugates were added to the cell culture medium 5 h after minigene transfection. Application of asPNA–neamine conjugates could restore the exon 10 inclusion level to close to 50%.

As expected, for the cells transfected with the +19G minigene alone, the exon 10 inclusion level was close to 0% (Figure 4, lanes 1 and 11). We then cotransfected cells with the +19G minigene and varied concentrations of asPNA(+8/+18). Significantly, upon the application of 1, 10, and 20 µM asPNA(+8/+18), the exon 10 inclusion level increased in a dose-dependent manner to 3%, 27%, and 59%, respectively (Figure 4, lanes 2–4). Note that our gel shift assay revealed a nM binding for asPNA(+8/+18). A relatively high concentration of asPNA(+8/+18) was needed for the observable regulatory effect in the cell culture, probably because the preformed splice site structure slows down the binding rate of asPNAs. It is also probable that a relatively low efficiency of cellular uptake of PNA reduces the cellular activity of asPNA(+8/+18). We observed no significant change for exon 10 inclusion upon the application of 20 µM asPNA(−8/+2), which is complementary to the 5′ arm of the hairpin (0%, Figure 4, lane 5).

We next tested whether asPNA binding to 5′ss may mask its recognition by U1 snRNP (Figure 1) and thus inhibit exon 10 inclusion. As expected, for the cells transfected with +14U minigene alone, the exon 10 inclusion level was close to 100% (Figure 4, lane 6). We then cotransfected the cells with +14U minigene and varied concentrations of asPNA(−8/+7). The exon 10 inclusion level decreased in a dose-dependent manner from 100% to 98%, 87%, and 81%, respectively, upon the application of 1, 10, and 20 µM asPNA(−8/+7) (Figure 4, lanes 7–9). Upon cotransfection with 20 µM of asPNA(−8/+2), which is a truncated version of asPNA(−8/+7), no significant change was observed in exon 10 inclusion (99%, Figure 4, lane 10). The result indicated that asPNAs targeting pre-mRNA residues between +2 and +7 may be critical in competing with U1 snRNA binding to pre-mRNA residues +1 to +7 (Figure 1a) and thus inhibiting exon 10 inclusion. It is also probable that asPNA(−8/+2) has a slightly weakened binding compared to asPNA(−8/+7) (Figure 3), resulting in no inhibition of exon 10 inclusion.

We next conjugated the asPNAs with an amino sugar neamine (see Figure 2j) to enhance cellular uptake and to avoid the use of a transfection reagent [71,72]. We attached neamine to the N-terminus of asPNA(+8/+18) to obtain a PNA–neamine conjugate, asPNA(+8/+18)–Nea. Five hours after minigene transfection, we applied asPNA(+8/+18)–Nea (method B). We observed that the application of 20 µM asPNA(+8/+18)–Nea resulted in the +19G minigene exon 10 inclusion level increasing from 0% to 56% (Figure 4, lane 12), which is comparable to the effect of cotransfecting nonconjugated asPNA(+8/+18). No significant change in the exon 10 inclusion level was observed upon the application of 20 µM asPNA(−8/+2)–Nea (0%, Figure 4, lane 13), indicating that neamine alone may not affect splicing. It is important to note that the covalent conjugation of PNAs with neamine avoids the use of transfection reagents and may be more advantageous for potential therapeutic applications.

Similarly, we conjugated neamine with asPNA(−8/+2), asPNA(−9/+3), asPNA(−9/+4), and asPNA(−8/+7) with varied lengths and target regions of the 5′ arm of the hairpin (Figure 2). Among the four asPNA–neamine conjugates tested (Figure 4, lanes 15–18), asPNA(−8/+7)–Nea reduced the +14U minigene exon 10 inclusion level most significantly (69%, lane 15), followed by asPNA(−9/+3)–Nea (85%, lane 16), asPNA(−9/+4)–Nea (87%, lane 17), and asPNA(−8/+2)–Nea (92%, lane 18). Note that the *K*_d_ values for asPNA(−8/+2) (12.4 nM), asPNA(−9/+3) (7.3 nM), asPNA(−9/+4) (3.4 nM), and asPNA(−8/+7) (2.0 nM) (Figure 3) are significantly below the concentration (20 µM) used in the cell culture splicing assay. Thus, we may expect that the differences in splicing modulation of the asPNAs result mainly from the differences in the binding sites. The data for both neamine-conjugated and nonconjugated asPNAs suggest that it is important to block 5′ss positions around +2 to +7 (U1 snRNA binding site ranging from residue +1 to +7, Figure 1a) as a targeting site for reducing exon 10 inclusion levels. Overall, our results show that neamine-conjugated asPNAs could enter cells and alter the splicing of exon 10 in a length- and position-dependent manner.

We tested whether the application of a PNA to the cells could bind to DNA and alter the expression levels of endogenous and/or minigene tau transcripts. We measured the total RNA levels using real-time PCR (Supplementary Figure S4). The tau transcript expression levels upon the application of PNAs did not change significantly compared to the untreated controls. The real-time PCR data are consistent with our nondenaturing PAGE data, which suggests that asPNA(−8/+7) does not bind to the fully complementary tau model DNA duplex encoding the tau pre-mRNA hairpin sequence (Supplementary Figure S3). Taken together, our results suggest that asPNAs alter the splicing of exon 10 via strand invasion of the pre-mRNA hairpins but not by binding to DNA.

## 3. Materials and Methods

### 3.1. General Methods and Synthesis of PNA Oligomers

Reverse-phase high-performance liquid chromatography (RP-HPLC) purified RNA and DNA oligonucleotides were purchased from Sigma-Aldrich, Singapore. The PNA monomers were purchased from ASM Research Chemicals (Hannover, Germany). PNA oligomers were synthesized manually using *tert*-Butyloxycarbonyl protecting group (Boc) chemistry via a solid-phase peptide synthesis (SPPS) protocol. Here, 4-methylbenzhydrylamine hydrochloride (MBHA·HCl) polystyrene resins were used. The loading value used for the synthesis of the oligomers was 0.3 mmol/g, and acetic anhydride was used as the capping reagent. Benzotriazol-1-yl-oxytripyrrolidinophosphonium hexafluorophosphate (PyBOP) and *N,N*-diisopropylethylamine (DIPEA) were used as the coupling reagent. The oligomerization of PNA was monitored through a Kaiser test. Cleavage of the PNA oligomers was done using a trifluoroacetic acid (TFA) and trifluoromethanesulfonic acid (TFMSA) method, after which the oligomers were precipitated with diethyl ether, dissolved in deionized water, and purified by reverse-phase high-performance liquid chromatography (RP-HPLC) using H_2_O–CH_3_CN–0.1% TFA as the mobile phase. Matrix-assisted laser desorption/ionization time-of-flight (MALDI-TOF) analysis was used to characterize the oligomers (Table S1, Supplementary Figure S1), with the use of α-cyano-4-hydroxycinnamic acid (CHCA) as the sample crystallization matrix.

### 3.2. Nondenaturing Polyacrylamide Gel Electrophoresis

Nondenaturing (12 wt%) polyacrylamide gel electrophoresis (PAGE) experiments were conducted with an incubation buffer containing 200 mM NaCl, 0.5 mM ethylenediaminetetraacetic acid (EDTA), and 20 mM 4-(2-hydroxyethyl)-1-piperazineethanesulfonic acid (HEPES) at pH 7.5. The concentration of RNA (labeled with Cy3 at the 5′ end) was 5 nM. The loading volume for samples containing RNA hairpins was 20 µL. The samples were prepared by snap cooling of the hairpins, followed by annealing with PNA oligomers by slow-cooling from 65 °C to room temperature and incubation at 4 °C overnight. Prior to loading the samples into the wells, 35% glycerol (20% of the total volume) was added to the sample mixtures. A running buffer containing 1× Tris–Borate–EDTA (TBE) buffer, pH 8.3, was used for all the gel experiments. The gel was run at 4 °C at 250 V for 5 h.

### 3.3. Cell Culture Minigene Splicing Assay

HEK293T cells were cultured in Hyclone Dulbecco′s Modified Eagle′s Medium (DMEM) (Thermo Scientific, Waltham, MA, USA) with 10% (*v/v*) fetal bovine serum (FBS) and antibiotics (100 U·mL^−1^ penicillin and 100 mg·mL^−1^ streptomycin). For each experiment, ~50% confluent HEK293T cells in 96-well plates were transfected with 0.1 µg of DNA per well, using 0.3 µL of X-tremeGENE 9 DNA Transfection Reagent (Roche, Basel, Switzerland) diluted in 10 µL of Hyclone Opti-MEM (Thermo Scientific, Waltham, MA, USA). Typically, tau minigene constructs were mixed with control plasmids in a 1:11 ratio, as previously reported [27,29].

Two methods were used to test the effects of PNAs on splicing. In method A, the PNAs were mixed with the minigene transfection mixture detailed above and incubated for 20 min prior to transfection. In method B, the PNAs were covalently attached with neamine. The PNA–neamine conjugates were added to the cell culture medium 5 h after minigene transfection.

Cells were harvested 48 h after minigene transfection, and the total RNA was extracted using a PureLink^®^ RNA Mini Kit (Life Technologies, Carlsbad, CA, USA). Residual DNA was removed by RQ1 RNase-Free DNaseI (Promega, Madison, WI, USA) digestion, and the RNA was ethanol-precipitated. The RNA was reverse-transcribed with Moloney Murine Leukemia Virus Reverse Transcriptase (New England Biolabs, Ipswich, MA, USA) according to the manufacturer′s instructions, with oligo-dT (18 T) as a primer.

Our Universal Minigene Vector (UMV) was used to clone and express the tau minigenes [27,29]. The UMV has two constitutive exons (exons 8 and 10) from the coenzyme A dehydrogenase C-4 to C-12 straight chain gene (*ACADM*) and multiple cloning sites in the middle of the intron to introduce the test exons along with splice sites and other regulatory sequences. The UMV-expressed cDNAs were amplified using a pcDNA.F-R primer pair (GAGACCCAAGCTGGCTAGCGTT and GAGGCTGATCAGCGGGTTTAAAC), which was complementary to the transcribed region of the UMV minigene upstream of the 5′ exon and downstream of the 3′ exon. The forward primer (pcDNA.F) was labeled on the 5′ end with 6-FAM (fluorescein) by the manufacturer (Integrated DNA Technologies, Coralville, IA, USA). Semiquantitative PCR was performed as previously described [27,29] using the fluorescent-labeled primers. The PCR products were separated by 10% native PAGE at 10 V/cm for 6 h and 30 min in 1× TBE buffer. The gels were scanned with Typhoon Trio (GE Healthcare Life Sciences, Chicago, IL, USA) using a 532-nm green laser and a 526-nm short pass filter at 600 V at normal sensitivity at 50-μm resolution. The gel bands were quantified to obtain the exon inclusion percentages from the experimental triplicates, as detailed in our previous work [27,29].

### 3.4. Real-Time PCR

Real-time PCR was set up in 96-well microplates in a 10-μL mixture containing 2 μL of the eight-fold diluted cDNA, 10 μL SYBR Select Master Mix (Life Technologies, Carlsbad, CA, USA), and 200 nM of each primer in the CFX96 Real-Time PCR System (Bio-Rad, Hercules, CA, USA). The following parameters were used: 95 °C for 3 min, 40 cycles of 95 °C for 20 s, 58 °C for 30 s, and 72 °C for 90 s. The fluorescence threshold values (Ct) were calculated using a thermocycler system software. Tau endogenous and minigene transcript levels were normalized to β-actin. The primers used were β-actin (forward: 5′-CCAGAGGCGTACAGGGATAG-3′; reverse: 5′-CCAACCGCGAGAAGATGA-3′), endogenous tau (forward: 5′-AGGGGATCGCAGCGGCTACA-3′; reverse: 5′-CAGGTCTGGCATGGGCACGG-3′), and tau minigene (forward: 5′-GTCTTCGAAGATGTGAAAGTGCC-3′; reverse: 5′-GAGGCTGATCAGCGGGTTTAAAC-3′). The endogenous tau and minigene tau primer designs were adapted as previously reported [29,73]. Fluorescence threshold values (Ct) were used to calculate relative mRNA expression by the 2-ΔΔCt relative quantification method, whereby the values were expressed as fold change over the corresponding values for the control. Three technical replicates for each of three biological replicates were performed.

## 4. Conclusions

We showed that relatively short asPNAs (10–15 mer) could invade the tau pre-mRNA exon 10 regulatory hairpin with nanomolar binding affinities. Cotransfection of asPNAs with a commercially available DNA transfection reagent could facilitate the cellular regulation of tau minigene alternative splicing. Furthermore, conjugation of asPNAs with neamine facilitated splicing regulation without a transfection reagent. The asPNAs did not invade the fully complementary DNA duplex, which was consistent with the fact that the application of the asPNAs did not affect the tau transcript levels. The findings indicate that asPNAs may be useful as probes and therapeutics targeting tau pre-mRNA exon 10 splicing.

Our work indicates that it is critical to mask or expose the residues (+2 to +7) adjacent to the tau pre-mRNA exon 10 5′ss by asPNAs for exon 10 exclusion (e.g., asPNA(−8/+7)) or inclusion (e.g., asPNA(+8/+18)). The 3′ arm of the hairpin may contain the binding sites for *trans*-acting intronic splicing silencer (ISS)-binding proteins [31,32,33,34], and thus the effect of asPNA binding to the 3′ arm of the hairpin (e.g., asPNA(+8/+18)) may also be due to the inhibition of the binding of *trans*-factors to the ISS. Consistently, an 18-mer antisense oligonucleotide complementary to the residues +11 to +28 showed in vivo activity in increasing exon 10 inclusion, probably due to the combined effects of exposing 5′ss and inhibiting *trans*-factors binding to ISS [16]. Interestingly, an 18-mer antisense oligonucleotide complementary to the residues +3 to +20 inhibited exon 10 inclusion [16], suggesting that the effect of the inhibition of U1 snRNP binding to the 5′ss dominated that of masking the ISS. However, two previously reported 25-mer antisense oligonucleotides complementary to the residues −10 to +15 and +2 to +26 showed no activity in regulating exon 10 inclusion [42], probably because the effects of inhibiting U1 snRNP binding to the 5′ss neutralized those of inhibiting *trans*-factor binding to the ISS and the adjacent intronic splicing modulator (ISM). Interestingly, a 21-mer antisense oligonucleotide complementary to the residues −8 to +13 inhibited exon 10 inclusion [38], which may have resulted from the combined effects of binding to the exonic splicing enhancer (ESS), the U1 snRNA recognition sequence, and the ISS. Clearly, one may take RNA secondary structure, splice site position, and binding of protein-splicing regulators into consideration for designing splicing modulating antisense compounds.

Our data provide important insights into developing ligands targeting the tau pre-mRNA hairpin structure. For example, double-stranded RNAs (dsRNAs) may be targeted by chemically modified dsRNA-binding PNAs that show significantly reduced binding to single-stranded RNAs (ssRNAs) [72,74,75,76,77,78] and dsDNAs [72,74,75,76,77,78,79,80,81,82,83]. However, the application of dsRNA-binding PNAs for regulating tau pre-mRNA exon 10 splicing may not be ideal, because the splice site hairpin contains a relatively short dsRNA region (seven base pairs), and residues +5 to +7 (critical for U1 snRNP recognition) are not involved in stable base pairing interactions [21,29,84]. Thus, one may target structured RNAs with structure-disrupting asPNAs or structure-recognizing ligands depending on the function of the RNA sequence and structure.

## Supplementary Materials

The Supplementary Materials are available online.
