# Supplementary material for: RNA Secondary Structure-Based Design of Antisense Peptide Nucleic Acids for Modulating Disease-Associated Aberrant Tau Pre-mRNA Alternative Splicing
Source: Molecules. 2019 Aug 20;24(16):3020. doi: 10.3390/molecules24163020 (PMC6720520; doi:10.3390/molecules24163020)
Supplement: Supplementary file 1 [file molecules-24-03020-s001.pdf]

Supporting Information

RNA Secondary Structure-Based Design of Antisense Peptide Nucleic Acids for Modulating Disease-Associated Aberrant Tau pre-mRNA Alternative Splicing

Alan Ann Lerk Ong [1,2,†], Jiazi Tan [2,†], Malini Bhadra [3], Clément Dezanet [4], Kiran M. Patil [2], Mei Sian Chong [5], Ryszard Kierzek [6], Jean-Luc Decout [4], Xavier Roca [3] and Gang Chen [2,*]

[1] NTU Institute for Health Technologies (HeathTech NTU), Interdisciplinary Graduate School, Nanyang Technological University, 50 Nanyang Drive, Singapore 637553, Singapore

[2] Division of Chemistry and Biological Chemistry, School of Physical and Mathematical Sciences, Nanyang Technological University, 21 Nanyang Link, Singapore 637371, Singapore

[3] School of Biological Sciences, Nanyang Technological University, Singapore 637551, Singapore

[4] University Grenoble Alpes/CNRS, Département de Pharmacochimie Moléculaire, ICMG FR 2607, UMR 5063, 470 Rue de la Chimie, F-38041 Grenoble, France

[5] Geriatic Education & Research Institute, 2 Yishun Central 2, Singapore 768024, Singapore

[6] Institute of Bioorganic Chemistry, Polish Academy of Sciences, Noskowskiego 12/14, 61-704 Poznan, Poland

* Correspondence: RNACHEN@ntu.edu.sg; Tel: +65 6592 2549; Fax: +65 6791 1961

† These authors contributed equally to this work.

Table of Contents

1. Table S1 and Figure S1: MALDI-TOF data S3-S8

2. Figures S2 and S3: Nondenaturing PAGE results S9-S10

3. Figure S4: Real-time PCR data S11-S12

2. MALDI-TOF of PNA oligomers

Table S1. Sequences and MALDI-TOF data for synthesized PNAs and PNA-neamine conjugates.

PNA	Sequence	Molecular Formula	Calculated MW	Observed MW
asPNA(+8/+18)	NH_2-Lys-ACGTGTGAAGG-$CONH_2$	$C_{126}H_{172}N_{72}O_{33}$	3211.3	3212.5
asPNA(-8/+2)	NH_2-Lys-ACACTGCCGC-$CONH_2$	$C_{111}H_{146}N_{60}O_{30}$	2799.2	2801.3
asPNA(-8/+7)	NH_2-Lys-TACTCACACTGCCGC-$CONH_2$	$C_{164}H_{213}N_{85}O_{46}$	4108.7	4109.7
asPNA(-9/+3)	NH_2-Lys-CACACTGCCGCC-$CONH_2$	$C_{131}H_{172}N_{70}O_{36}$	3301.4	3302.4
asPNA(-9/+4)	NH_2-Lys-TCACACTGCCGCC-$CONH_2$	$C_{142}H_{186}N_{74}O_{40}$	3567.5	3568.6
asPNA(+8/+18)-Nea	NH_2-Nea-Lys-ACGTGTGAAGG-$CONH_2$	$C_{148}H_{203}N_{77}O_{41}$	3714.6	3717.2
asPNA(-8/+2)-Nea	NH_2-Nea-Lys-ACACTGCCGC-$CONH_2$	$C_{133}H_{187}N_{65}O_{38}$	3302.5	3303.5
asPNA(-8/+7)-Nea	NH_2-Nea-Lys-TACTCACACTGCCGC-$CONH_2$	$C_{186}H_{254}N_{90}O_{54}$	4612.0	4616.9
asPNA(-9/+3)-Nea	NH_2-Nea-Lys-CACACTGCCGCC-$CONH_2$	$C_{153}H_{213}N_{75}O_{44}$	3804.7	3805.7
asPNA(-9/+4)-Nea	NH_2-Nea-Lys-TCACACTGCCGCC-$CONH_2$	$C_{164}H_{227}N_{79}O_{48}$	4070.8	4071.9

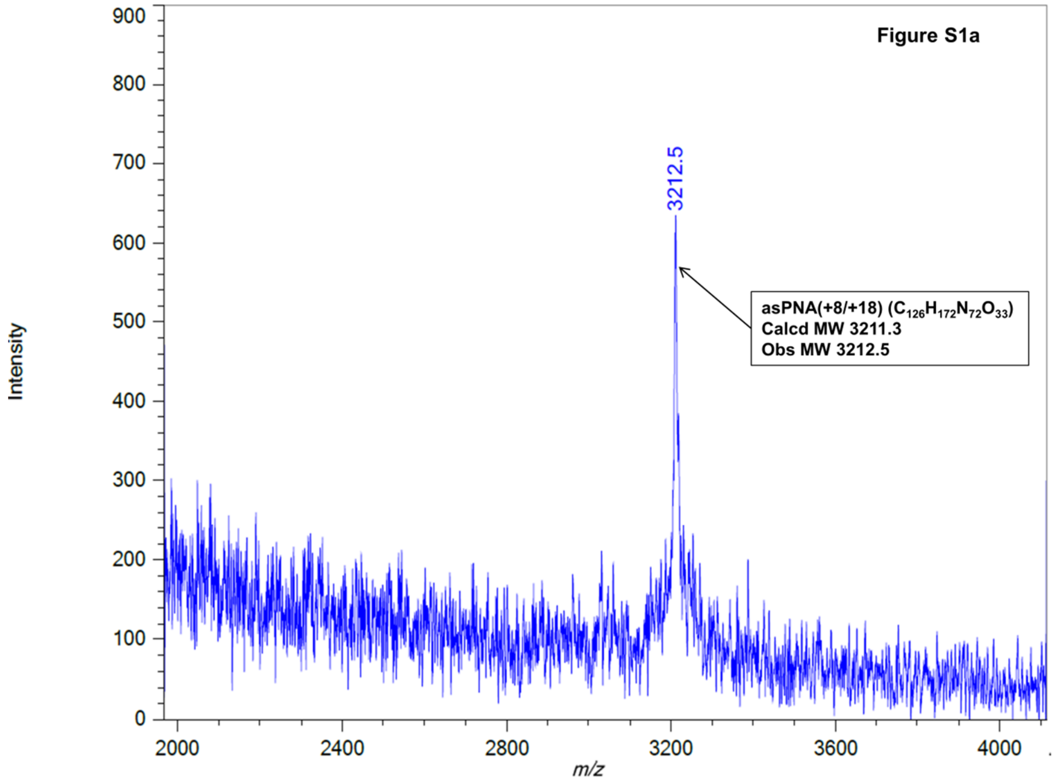
Figure S1a
asPNA(+8/+18) ($C_{126}H_{172}N_{72}O_{33}$)
Calcd MW 3211.3
Obs MW 3212.5
3212.5
Intensity
m/z

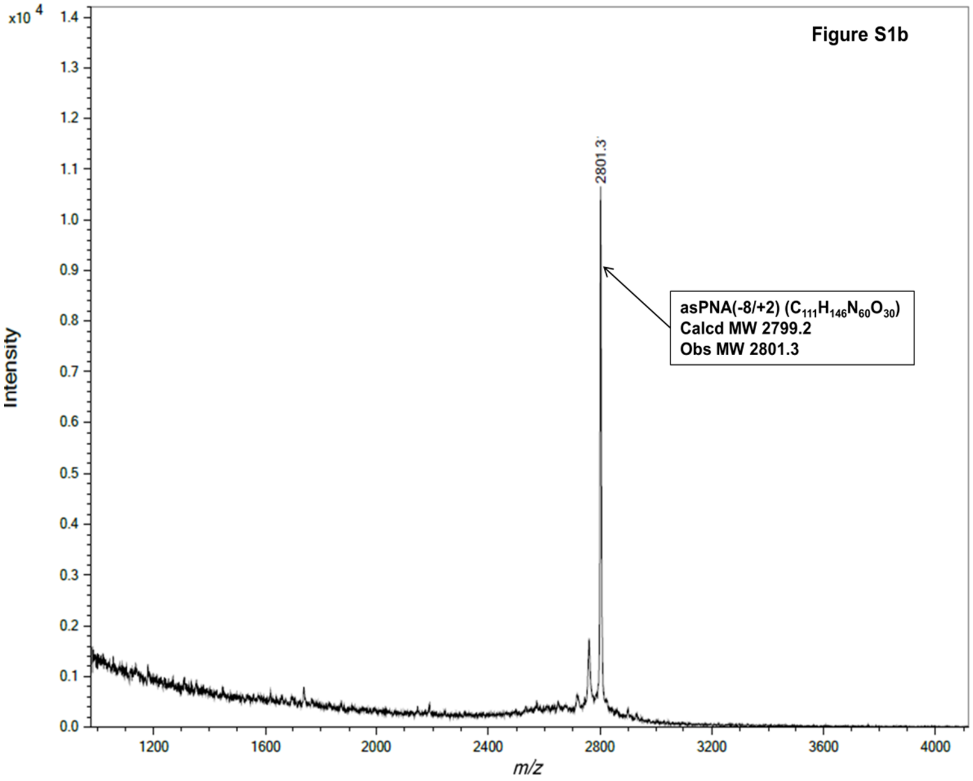
Figure S1b
asPNA(-8/+2) ($C_{111}H_{146}N_{60}O_{30}$)
Calcd MW 2799.2
Obs MW 2801.3
2801.3
x10 4
Intensity
m/z

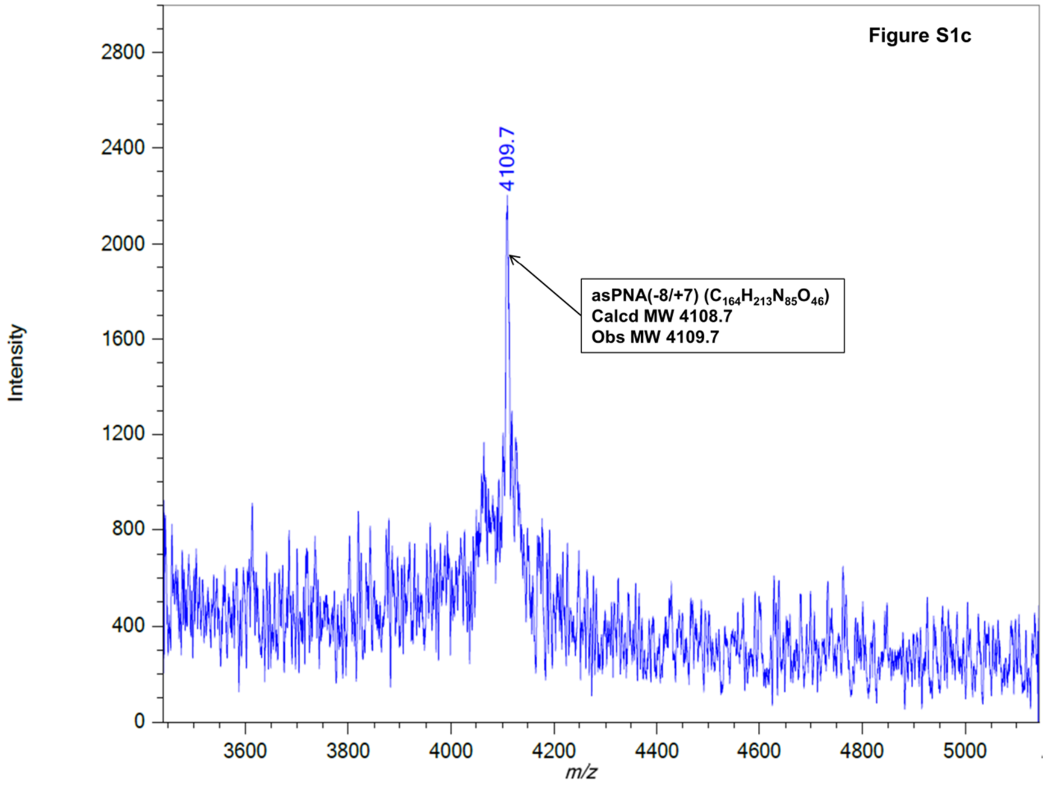
Figure S1c
asPNA(-8/+7) ($C_{164}H_{213}N_{85}O_{46}$)
Calcd MW 4108.7
Obs MW 4109.7
4109.7
Intensity
m/z

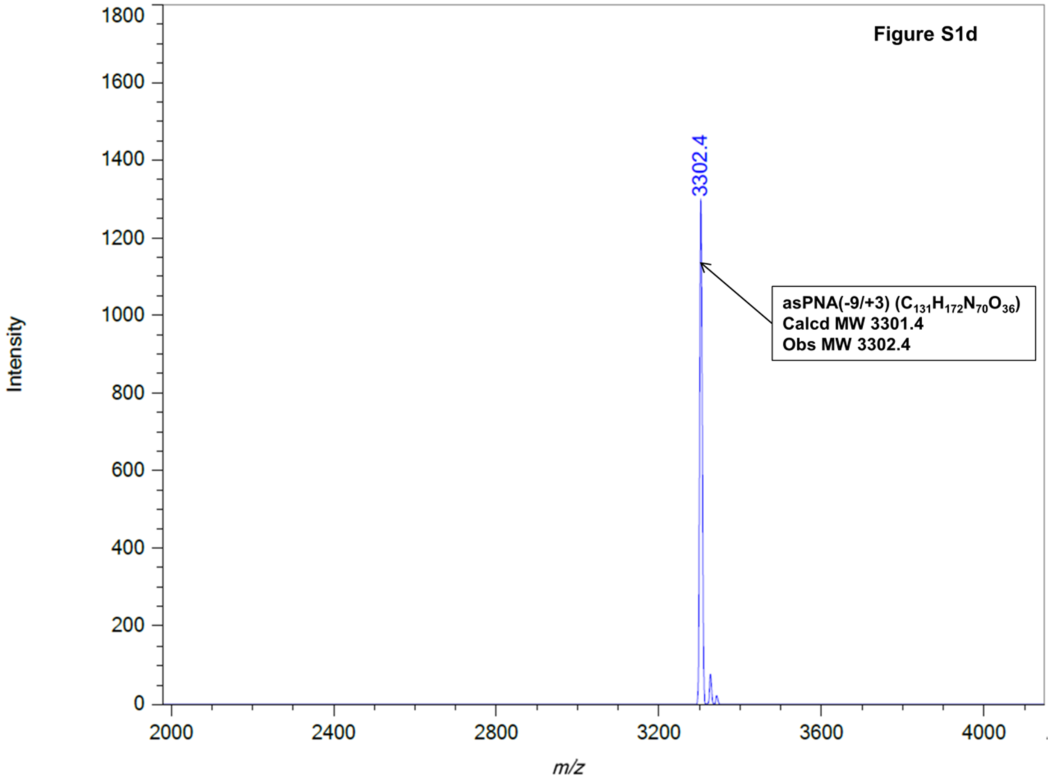
Figure S1d
asPNA(-9/+3) ($C_{131}H_{172}N_{70}O_{36}$)
Calcd MW 3301.4
Obs MW 3302.4
3302.4
Intensity
m/z

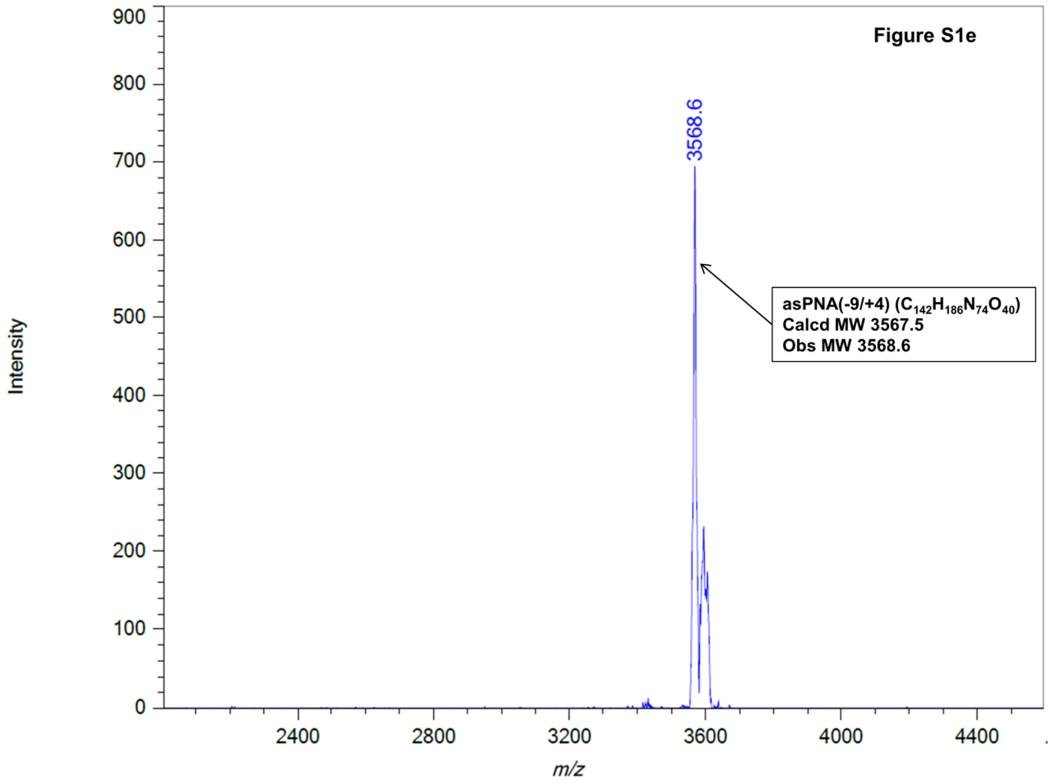
Figure S1e
3568.6
asPNA(-9/+4) ($C_{142}H_{186}N_{74}O_{40}$)
Calcd MW 3567.5
Obs MW 3568.6
Intensity
m/z

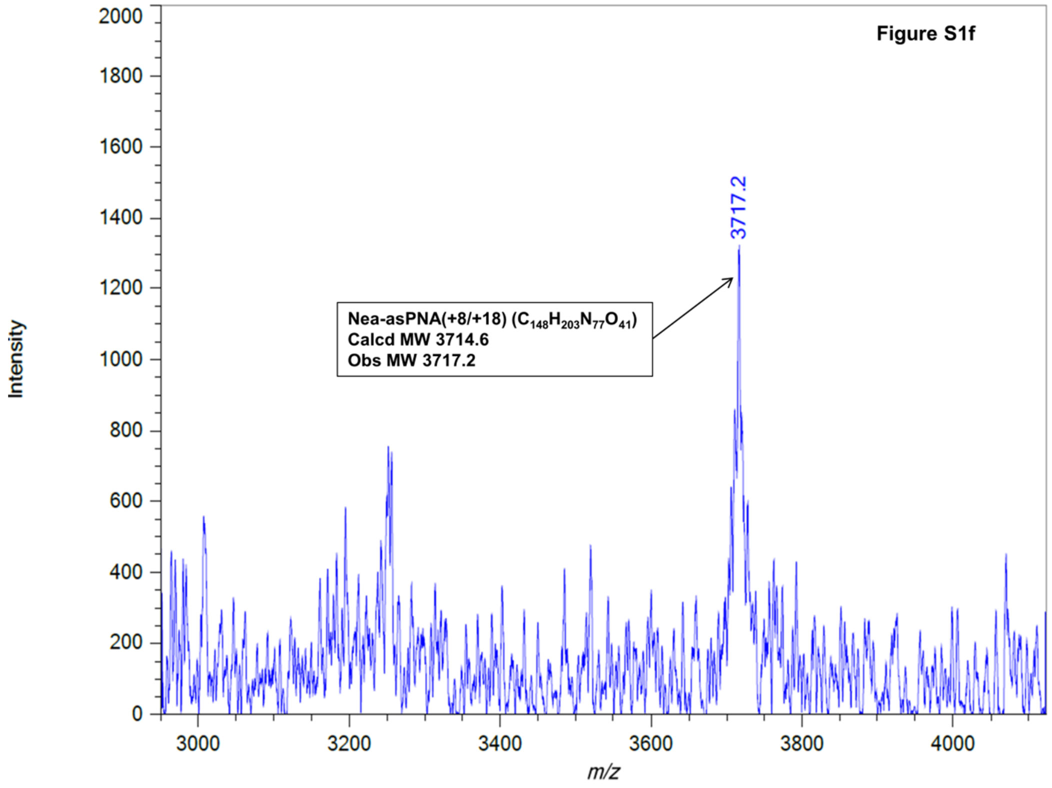
Figure S1f
3717.2
Nea-asPNA(+8/+18) ($C_{148}H_{203}N_{77}O_{41}$)
Calcd MW 3714.6
Obs MW 3717.2
Intensity
m/z

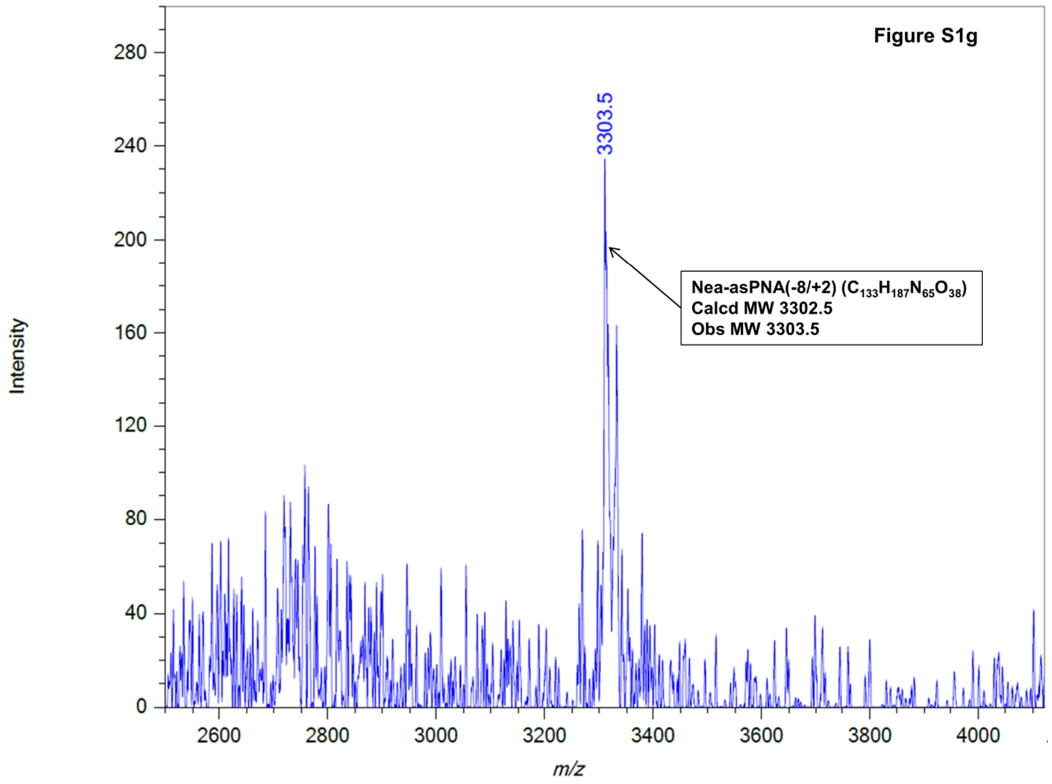

Figure S1g
3303.5
Nea-asPNA(-8/+2) ($C_{133}H_{187}N_{65}O_{38}$)
Calcd MW 3302.5
Obs MW 3303.5
Intensity
0
40
80
120
160
200
240
280
2600
2800
3000
3200
3400
3600
3800
4000
m/z

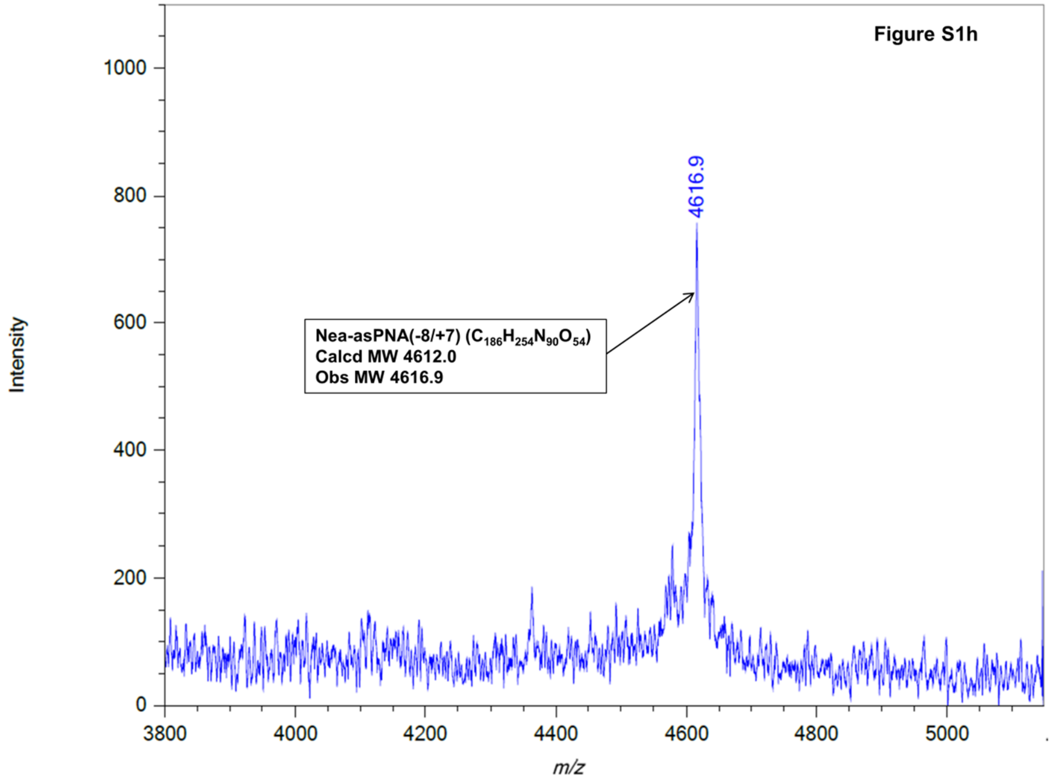

Figure S1h
4616.9
Nea-asPNA(-8/+7) ($C_{186}H_{254}N_{90}O_{54}$)
Calcd MW 4612.0
Obs MW 4616.9
Intensity
0
200
400
600
800
1000
3800
4000
4200
4400
4600
4800
5000
m/z

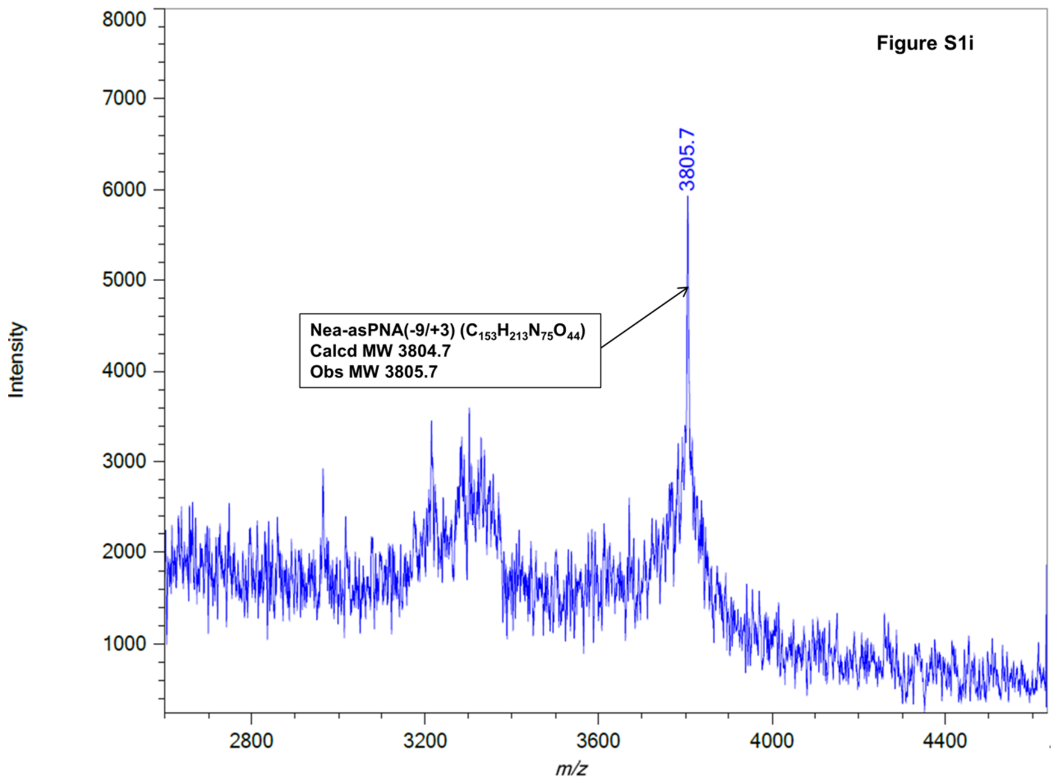

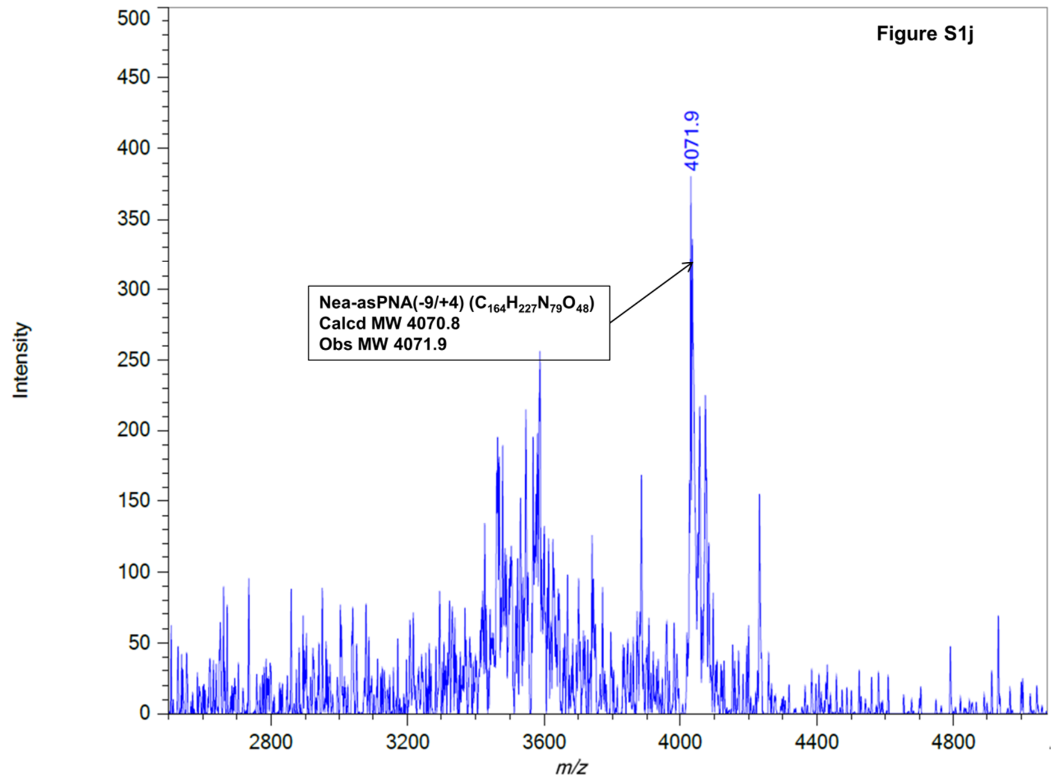

Figure S1. MALDI-TOF data of PNAs and PNA-neamine conjugates studied in this paper.

3. Non-denaturing PAGE results

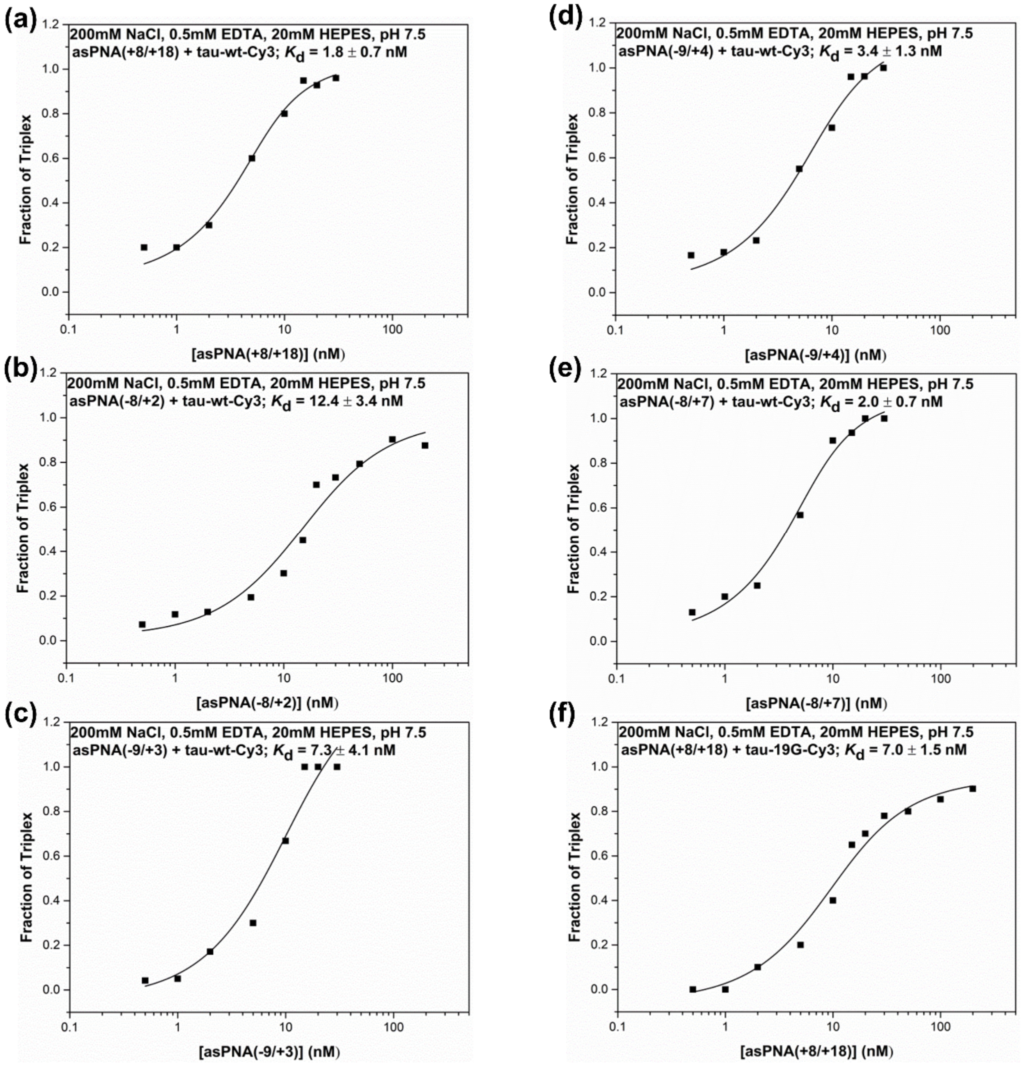

Figure S2. K_d determination by nondenaturing PAGE for various PNAs binding to tau pre-mRNA hairpins. The fraction of RNA-PNA complex formation was calculated based on the band intensities. The data were fit to the equation: $Y = Y_0 + (B/2/R_0)(R_0 + X + K_d - ((R_0 + X + K_d)^2 - 4R_0X)^{1/2})$ where R_0 is the RNA hairpin concentration (5 nM). Y_0 and B are the minimum and maximum fraction of triplex formation respectively. X is the total PNA concentration and K_d is the dissociation constant. For panels a, c-e, the points at the highest concentrations (50, 100 and 200 nM) are removed to avoid over fitting of the high-concentration base lines.

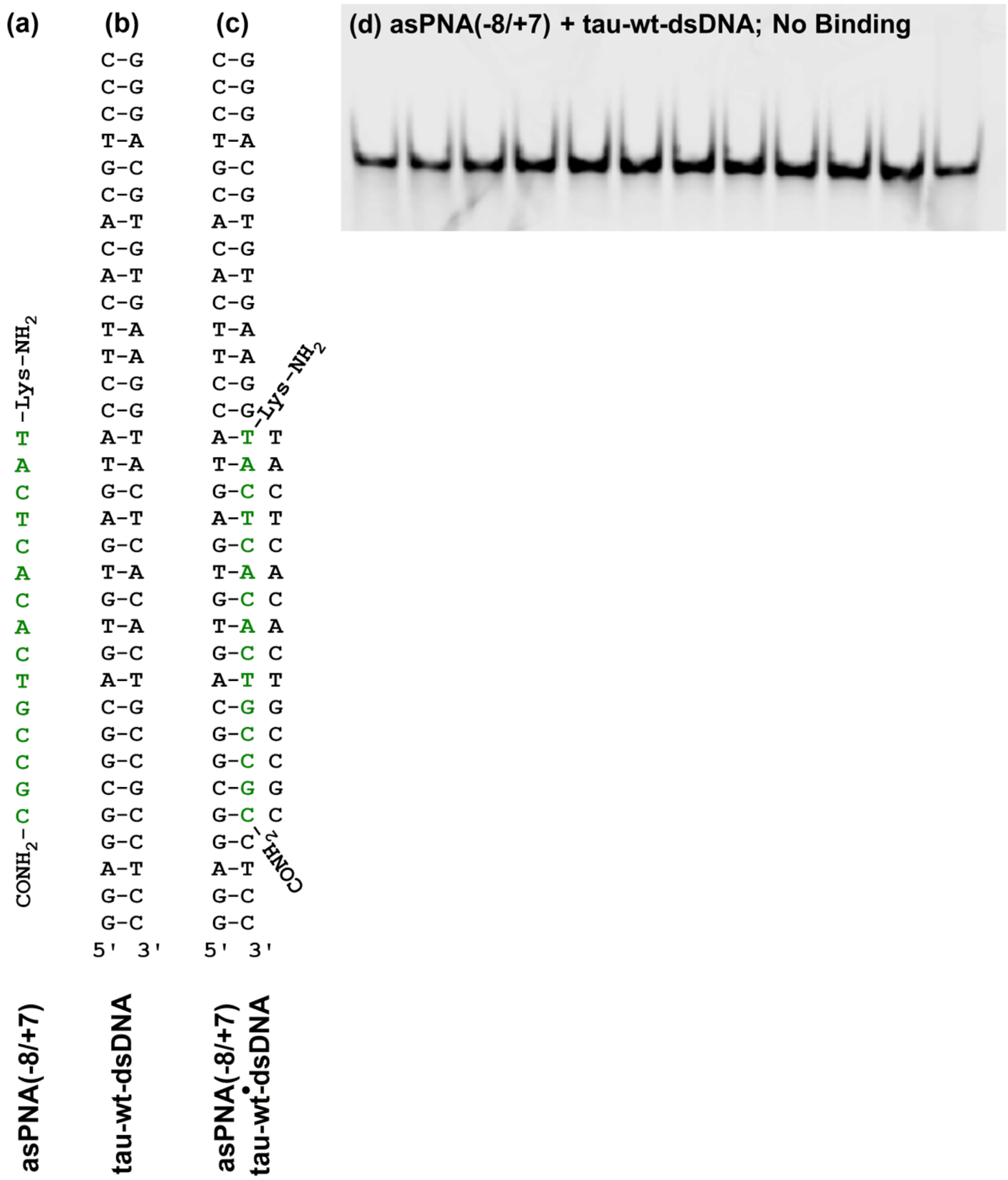

Figure S3. Nondenaturing PAGE assay for asPNA binding to DNA duplex. **(a-c)** Structures of asPNA(-8/+7), the model tau wild-type DNA duplex (tau-wt-dsDNA) encoding tau pre-mRNA exon 10 5′ss site hairpin, and potential invasion complex formed between PNA and DNA duplex. **(d)** Nondenaturing PAGE for asPNA(-8/+7) binding to tau-wt-dsDNA. The incubation buffer contains 200 mM NaCl, 0.5 mM EDTA, 20 mM HEPES, pH 7.5. The loaded samples contain 1 μM of DNA hairpin in 20 μL. The PNA concentrations in lanes from left to right are: 0, 0.2, 0.4, 1, 1.6, 2, 4, 10, 16, 20, 28, and 50 μM. The gel result suggests that asPNA(-8/+7) does not bind to DNA duplex at this condition.

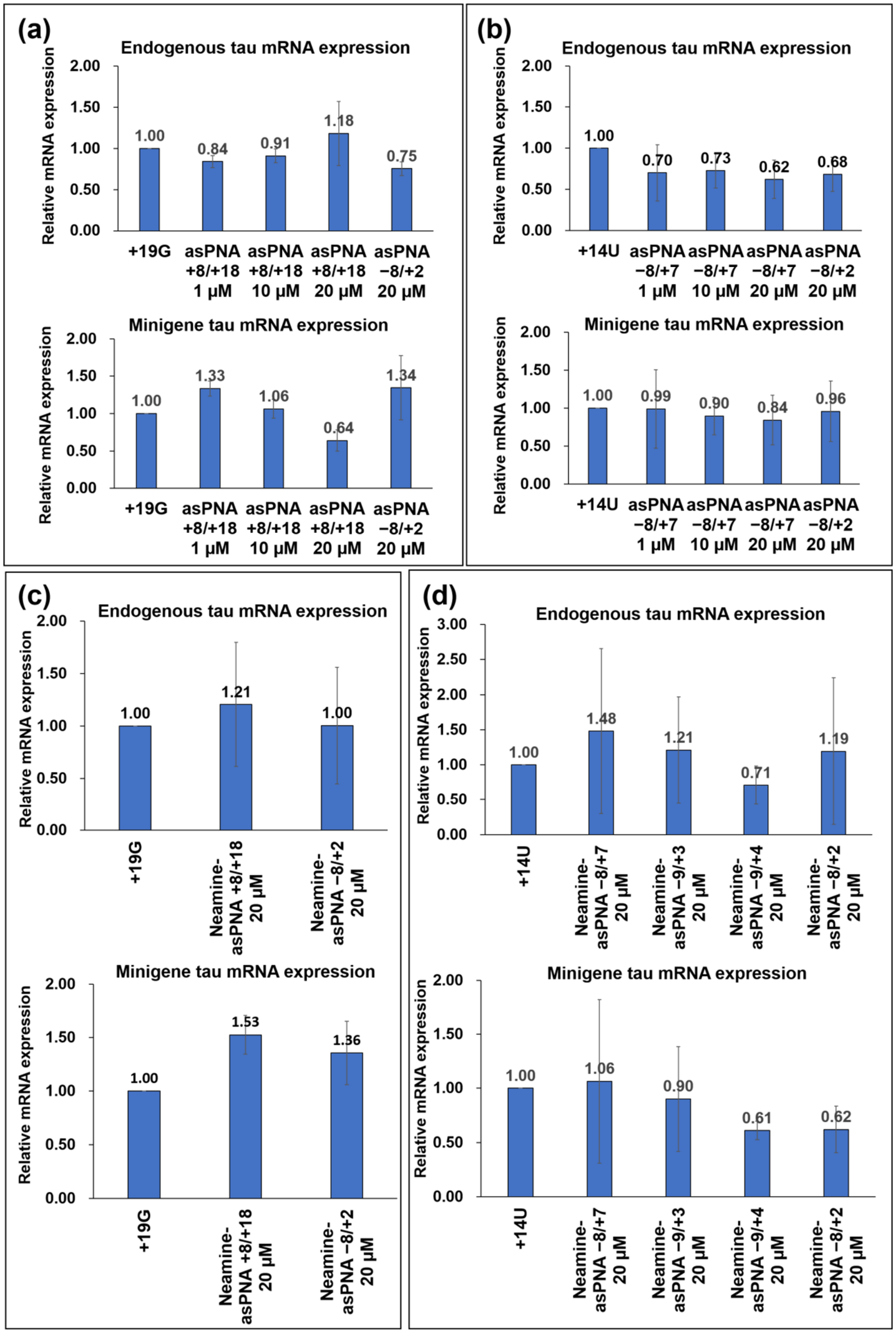

Figure S4: Real-time PCR data showing the transcript levels of endogenous tau and minigenes. (a) Co-transfection of +19G minigene with asPNAs (method A). (b) Co-transfection of minigene +14U and asPNAs (method A). (c) Transfection of minigene +19G, followed by the application of asPNA-neamine conjugates (method B). (d)

Transfection of minigene +14U, followed by the application of asPNA-neamine conjugates (method B). The experiments were performed in triplicates and the data are shown as mean ± standard deviation. Student's t-test was performed with adjusted P-values using Bonferroni correction [$P = 0.0125$ for (a), (b), (d); $P = 0.025$ for (c)].
